# Age-Related Decreases in Interhemispheric Resting-State Functional Connectivity and Their Relationship With Executive Function

**DOI:** 10.3389/fnagi.2020.00020

**Published:** 2020-02-26

**Authors:** Jizheng Zhao, Peter Manza, Corinde Wiers, Huaibo Song, Puning Zhuang, Jun Gu, Yinggang Shi, Gene-Jack Wang, Dongjian He

**Affiliations:** ^1^College of Mechanical and Electronic Engineering, Northwest A&F University, Yangling, China; ^2^Key Laboratory of Agricultural Internet of Things, Ministry of Agriculture, Yangling, China; ^3^Shaanxi Key Laboratory of Agricultural Information Perception and Intelligent Service, Yangling, China; ^4^Laboratory of Neuroimaging, National Institute on Alcohol Abuse and Alcoholism, Bethesda, MD, United States; ^5^Department of Endocrinology, First Affiliated Hospital of Hebei North University, Zhangjiakou, China

**Keywords:** executive function, voxel-mirrored homotopic connectivity, Delis-Kaplan executive function system, mediation analysis, medial temporal lobe subsystem, salience network, frontoparietal control network

## Abstract

Age-related alterations of functional brain networks contribute to cognitive decline. Current theories indicate that age-related intrinsic brain functional reorganization may be a critical marker of cognitive aging. Yet, little is known about how intrinsic interhemispheric functional connectivity changes with age in adults, and how this relates to critical executive functions. To address this, we examined voxel-mirrored homotopic connectivity (VMHC), a metric that quantifies interhemispheric communication, in 93 healthy volunteers (age range: 19–85) with executive function assessment using the Delis-Kaplan Executive Function System (D-KEFS) scales. Resting functional MRI data were analyzed to assess VMHC, and then a multiple linear regression model was employed to evaluate the relationship between age and the whole-brain VMHC. We observed age-related reductions in VMHC of ventromedial prefrontal cortex (vmPFC) and hippocampus in the medial temporal lobe subsystem, dorsal anterior cingulate cortex and insula in salience network, and inferior parietal lobule in frontoparietal control network. Performance on the color-word inhibition task was associated with VMHC of vmPFC and insula, and VMHC of vmPFC mediated the relationship between age and CWIT inhibition reaction times. The percent ratio of correct design scores in design fluency test correlated positively with VMHC of the inferior parietal lobule. The current study suggests that brain interhemispheric functional alterations may be a promising new avenue for understanding age-related cognitive decline.

## Introduction

Cognitive function is altered with age (Ulman, 2014; Harrington et al., 2017). In particular, its decline with age affects quality of life and life satisfaction in older adults (Reuter-Lorenz and Park, 2010; Ferreira and Busatto, 2013). Executive functions broadly consist of inhibition, working memory, and cognitive flexibility (Diamond, 2013). Studies on cognitive performance in healthy elder groups have indicated that increasing age is associated with multifaceted impairments of executive function (Ulman, 2014; Harrington et al., 2017). For example, aging is associated with impairment in cognitive performance in verbal fluency, category fluency, and category switching tests (Harrington et al., 2017). In addition, many studies have used the color-word Stroop task and found that response inhibition performance, or the ability to stop unwanted or inappropriate responses, declines with age (Ivnik et al., 1996; Klein et al., 1997; Troyer et al., 2006; Adólfsdóttir et al., 2016; Anderson and Craik, 2017; Harrington et al., 2017).

Resting-state functional MRI (rs-fMRI) imaging technology permits studying age-related intrinsic brain alterations *in vivo*. Accumulating studies have shown age-related regional functional connectivity (FC) decreases in brain regions within default mode (DMN), salience (SN), and frontoparietal control (FPCN) networks (Grady, 2012; Li et al., 2015). The most common FC reductions within DMN have been reported in medial prefrontal cortex and posterior cingulate cortex (PCC/precuneus) (Bluhm et al., 2008; Damoiseaux et al., 2008; Koch et al., 2010; Allen et al., 2011; Wu et al., 2011; Tomasi and Volkow, 2012; Grady et al., 2016). Age increase is also generally associated with decreases in intra-network FC within the bilateral insula and dorsal anterior cingulate cortex (dorsal ACC) (Keiichi et al., 2012; He et al., 2014; Zhang et al., 2014). FPCN shows numerically lower intra-network FC in older adults compared to young adults (Elman et al., 2014; Geerligs et al., 2014a; Grady et al., 2016). Further, the FC reduction within FPCN has been shown in middle-aged (41–60 years) compared to young (21–40 years) individuals (Siman-Tov et al., 2017). The reduced network covariation is in line with the idea that increasing age is accompanied by decreasing connectivity within functional brain systems (Chan et al., 2014; Geerligs et al., 2014a; Grady et al., 2016). Moreover, inter-network FC patterns have shown alterations with aging, including reductions in the segregation of DMN, SN, and FPCN (Chan et al., 2014; Geerligs et al., 2014a), and enhancements in FC strength between DMN and FPCN with age (Geerligs et al., 2014a; Grady et al., 2016). Thus, rs-fMRI may be a powerful tool to investigate age-related brain functional reorganization.

Neuropsychology studies have indicated that the age-related intrinsic functional reorganizations of DMN, SN, and FPCN are associated with impaired executive function. Age-related reductions in FC between MPFC and PCC/precuneus correlated with loss of executive function, memory, and processing speed (Andrews-Hanna et al., 2007). A higher number of Stroop errors correlated with reduced FC within the DMN and SN in cognitively normal elders (Duchek et al., 2013). In older adults, the strength of network covariation of the left insula and dorsal ACC in SN correlated significantly with executive functions measured by Frontal Assessment Battery (FAB) and Kohs Block-Design Test (Keiichi et al., 2012). Further, FC between SN and frontal cortex successfully predicted response inhibition as assessed by the Stroop test (La Corte et al., 2016). The between-network connectivity of the FPCN is enhanced in older subjects, and its strength is positively correlated with associative memory performance (Grady et al., 2016). These findings on intrinsic brain functional reorganizations shed light on the neural mechanisms underlying age-related executive function decline.

Theories on the relationship between age and neurocognition suggest a hemispheric asymmetry reduction for older adults (HAROLD model) in response to cognitive tasks (Cabeza, 2002; Manuela et al., 2013). For example, elders were shown to recruit a more bilateral frontal pattern within the task-related network to achieve successful performance during working memory (Sala-Llonch et al., 2012) and inhibitory control (Colcombe et al., 2005) tasks, while younger groups recruited the right-lateralized frontal regions (Colcombe et al., 2005; Sala-Llonch et al., 2012). This reduced lateralization pattern in frontal cortex suggests that functional reorganization occurs across hemispheres with age, and therefore, these changes may be measurable outside of the task state (e.g., alterations in resting-state FC or in structural changes). In support of this idea, a study on white matter integrity has shown that age-related changes are prominently seen in the anterior corpus callosum (Frederiksen and Waldemar, 2012), which is involved in information transformation across the right and the left brain hemisphere. For example, the changes of anterior corpus callosum are suggested to be accompanied by alterations of interhemispheric FC pattern of frontal cortex for healthy young participants (Qiu et al., 2017). However, little is known about the age-related alterations of interhemispheric FC pattern and whether such interhemispheric functional alterations contribute to age-related executive function change.

In the rs-fMRI literature, voxel-mirrored homotopic connectivity (VMHC) offers a metric to evaluate interhemispheric FC (Zuo et al., 2010), which measures integrity of information communication between brain hemispheres. Abnormal VMHC patterns in widespread cortical and subcortical networks have been reported in studies on cocaine addiction (Kelly et al., 2011), mild cognitive impairment (MCI) (Luo et al., 2018), Alzheimer’s disease (Wang et al., 2015; Li et al., 2018), and schizophrenia (Hoptman et al., 2012), indicating that VMHC is a reliable neural marker for brain functional reorganization. This abnormal VMHC has been associated with impaired executive functioning in individuals with MCI (Luo et al., 2018) and Alzheimer’s disease (Li et al., 2018), suggesting that altered VMHC might associate with executive function change. Taken together, the VMHC-based rs-fMRI analysis may provide additional information beyond classical FC metrics for understanding neural mechanisms of age-related executive function alteration. To our best knowledge, only one study has explored the relationship between VMHC and age (Zuo et al., 2010). This study included 7- to 85-year-old healthy participants, and focused on the developmental trajectories of brain inter-hemisphere FC in the lifespan (Zuo et al., 2010). However, the age-related homotopic FC alterations in adult and its associations with executive functions have not yet been examined.

We examined whether homotopic FC measured with VMHC changes with age in adults aged 19–85. Furthermore, we tested whether any age-related alterations of VMHC would be associated with executive function, as assessed by Delis-Kaplan Executive Function System (D-KEFS) scales. Finally, we employed a mediation analysis to identify whether interhemispheric connectivity is a possible neural mechanism underlying age-related cognitive decline.

## Materials and Methods

### Participants

For the current study, Nathan Klein Institute (NKI) data (Nooner et al., 2012) (demographic and executive function data, resting functional and structural MRI images) were downloaded from http://fcon_1000.projects.nitrc.org/indi/pro/nki.html. The NKI data included 207 subjects. First, subjects with a history of psychiatric disorders or medical conditions were excluded. For example, subjects with Beck Depression Inventory (BDI) (Beck et al., 1988) scores higher than 15, indicating mild-severe depression, were excluded. Forty-one healthy children and adolescents (age < 18) were excluded from the current study. For the 105 healthy adults, there were two subjects without D-KEFS scores (Homack et al., 2005) and three subjects without resting functional MRI data. Six subjects were excluded due to large head movements (mean framewise displacement >0.4 mm) (Power et al., 2014). One subject with extensive large color-word inhibition scores in D-KEFS test (105, which exceeded three standard deviations from the mean) was excluded. The final sample consisted of 93 healthy adults subjects (female: 45, male: 48; age range: 19–85, mean = 42.65 ± 1.93 SE years; 31 subjects aged 19–29, 11 subjects aged 30–39, 22 subjects aged 40–49, 8 subjects aged 50–59, 9 subjects aged 60–69, 9 subjects aged 70–79, 3 subjects aged 80–85) who completed resting-state MRI, structural scans, and the D-KEFS test. For the final sample, there was no subject with hypertension or diabetes, and systolic blood pressure was less than 140 mmHg. There was no subject taking daily medications. There was no subject with past or current mental disorder or substance abuse disorder. Handedness was assessed with the Edinburgh Handedness Inventory (Oldfield, 1971), height and weight of participant were measured on the day before the MRI scan, and then BMI was calculated. Table 1 provided the subjects’ demographic information.

**TABLE 1:** Demographic characteristics.

	**Range**	**Mean ± SE**
Age (years)	19–85	42.65 ± 1.93
Gender	F45/M48	
Handedness	Right 79/Left 12, one subject was unknown, one subject was ambidextrous	
BMI (kg/m^2^)	16.3–40	26.16 ± 0.50
BDI_Total	0–12	2.43 ± 0.33

### Executive Function Measurements

For the NKI data, five tests from D-KEFS were conducted to assess executive function, namely, Color-Word Interference (CWIT), Verbal Fluency, Design Fluency, Sorting, and Twenty Questions Tests. Detailed task descriptions can be found in Swanson (2005) and Mace et al. (2018). In brief, CWIT required participants to name the color or word in congruent and incongruent conditions. For example, when the word “green” was printed in red ink, participants were asked to process task-relevant color information (ink) and inhibit pre-potent processing of conflicting task-irrelevant information (word meaning). CWIT data included scores of word naming, color naming, and color-word inhibition. For the Verbal Fluency Test, participants were asked to name uniquely as many words as possible in 60 s by letter or category for altering categories. Data included scores of letter fluency, category fluency, category switching fluency, and category switching accuracy. On the Design Fluency Test, participants were instructed to draw unique geometric designs in dots arrays within 60 s. The paradigm consists of three conditions: connection of filled dots, connection of empty dots, and alternating connections between filled and unfilled dots. Data included scores of filled dots design, empty dots design, switching design, and total percent ratio of correct design. The Sorting Test asked participants to sort items into categories and describe the applied categorization rules and included two card sets. The number of correct sorts was computed and included scores of free sort confirmed sorts, free sort description, and sort recognition description. Twenty Questions Test asked participants to guess the objects from 30 common objects, and the participants were instructed to ask as few “yes” or “no” questions as possible. It included scores of initial abstraction, total questions asked, and total weighted achievement. The MRI scans were conducted at 9:00 am, and D-KEFS test was conducted at 12:30 pm on the same day.

### MRI Data

All participants provided written informed consent and were scanned according to procedures approved by the local Institutional Review Board (IRB) at the NKI. The data were shared with the approval of the IRB at the NKI. All subjects gave written informed consent in accordance with the Declaration of Helsinki.

Resting functional images were acquired using a Siemens MAGNETOM Tim Trio 3.0 T scanner. There were 260 functional MRI images (lasting for 10.83 min) with a gradient echo-planar sequence (TR = 2.5 s; TE = 35 ms; flip angle = 80°; FOV: 256 × 256; in-plane resolution = 3 mm × 3 mm, slice thickness: 3 mm). Structural MRI scans were acquired with the same Siemens MAGNETOM Tim Trio 3.0 T scanner using T1-weighted MPRAGE sequence (TR = 2.5 s; TE = 3.5 ms; TI = 1200 ms; FOV: 256 × 256; slice thickness: 1 mm; flip angle: 8°; matrix size: 256 × 256; 200 Transverse slices).

### MRI Preprocessing

Functional data were analyzed using Data Processing & Analysis for Brain Imaging (DPABI) toolbox (Yan et al., 2016). Image preprocessing included slice-time correction, image realignment, skull stripping, coregistration between functional and structural images, spatial normalization to the stereotactic space of the Montreal Neurological Institute (MNI), and resampling to 3-mm isotropic voxels and smoothing with a Gaussian kernel of 6-mm FWHM. Head motion correction was conducted based on a “scrubbing” approach (Power et al., 2014). Specifically, if the framewise displacement (FD) was larger than 0.5 mm, the corresponding volume was linearly interpolated using its temporal neighbors (Power et al., 2014). In addition, the mean FD value was used as a regressor in the group-level regression model and partial correlation analyses to control the possible motion influence. Multiple linear regression was performed to remove nuisances including the mean signal fluctuations in the whole brain, ventricles, and white matter, and the six head realignment parameters and their derivatives. Detrending and a temporal band-pass filtering (0.01–0.08 Hz) were subsequently conducted to minimize temporal drifts and white noise.

VMHC measurements assumed symmetric morphology between each brain hemisphere. To minimize differences in the geometric configuration of the cerebral hemispheres, we averaged 93 normalized T1 images to create a group-mean T1 image. This image was averaged with its left-right mirrored version to generate a group-specific symmetrical template. Each individual T1 image was non-linearly registered to the standard template, and the identical transformation was then applied to the resting-state functional images. VMHC was obtained by calculation of Pearson’s correlation coefficient between the time series of each voxel and that of its symmetrical interhemispheric counterpart. Voxels medial of *x* = ±4 were excluded, to minimize the blurring effect across the midline (Kelly et al., 2011).

### Correlation Analysis of D-KEFS Scores and Age

Partial correlations were conducted on each D-KEFS score and age, with gender, handedness, and body mass index (BMI) as covariates. Bonferroni correction was carried out for multiple comparisons, and level of significance was set at *P* < 0.0025 (0.05/18 for 18 D-KEFS scores). Finally, scores of color-word inhibition, category switching fluency and category switching accuracy, the percent ratio of correct design, and sort recognition description were significantly correlated with age. Then, the correlations of these five scores and age-related interhemispheric FC were examined.

### Statistical Analyses on Resting fMRI Data

Statistical analyses were performed using SPM12 (Welcome Department of Cognitive Neurology, London, United Kingdom)^1^. Multiple linear regression model was used to assess the association between age and VMHC metrics. The subject’s BMI, gender, handedness, mean FD, and total intracranial volume (TICV) were included as covariates. Statistical significance was based on a familywise error (FWE) correction for multiple comparisons at the cluster level (*P*_FWE_ < 0.05) with a minimum cluster size of *k* = 30 voxels and a cluster-defining threshold *P* < 0.001, in line with current reporting guidelines (Eklund et al., 2016; Flandin and Friston, 2019).

### ROI-Based Analysis

When the significant age-related statistics brain mapping was acquired, regions of interest (ROI) were defined by spheres with 6-mm radius and center at the local peak voxel in statistics brain mapping. Mean regional values were calculated for each subject. Then, group-level partial correlations were conducted on age-related regional VMHC values and scores of color-word inhibition, category switching fluency and category switching accuracy, the percent ratio of correct design, and sort recognition description respectively, with BMI, gender, handedness, mean FD, and TICV as covariates. Bonferroni correction was carried out for multiple comparisons, and level of significance was set at *P* < 0.0008 (0.05/12/5 for 12 pairs of mirrored regions in VMHC results by five D-KEFS scores).

In order to test whether shifting with one TR lag of time courses from the opposite hemisphere affected VMHC, Pearson correlation coefficients were calculated between time courses of brain regions in the left hemisphere lagging one TR and time courses of their mirrored brain regions in the right hemisphere without lag (termed FCs_leftlag), between time courses of brain regions in the left hemisphere without lag and time courses of their mirrored brain regions in the right hemisphere lagging one TR (termed FCs_rightlag), as well as between time courses of brain regions in the bilateral hemisphere both without lag (termed FCs_nolag). Three two-way ANOVA of FC types (specially, FCs_leftlag and FCs_nolag, FCs_rightlag and FCs_nolag, FCs_leftlag and FCs_rightlag) by region (12 pairs of mirrored brain regions) were conducted to examine the effect of time course lag.

### Mediation Analysis

For all age-related brain regions, we examined whether interhemispheric functional coupling of these regions mediated the relationship between age and these five scores respectively. We tested our mediation hypothesis with Multilevel Mediation and Moderation Toolbox (Wager et al., 2008), with age, BMI, gender, handedness, FD, and TICV as covariates. Bonferroni-correction was carried out for multiple comparisons, and level of significance was set at *P* < 0.0008 (0.05/12/5 for 12 pairs of mirrored regions in VMHC results by five D-KEFS scores).

## Results

### Behavioral Results

Table 2 shows the range, mean, and standard error of 18 D-KEFS scores, as well as their partial correlations with age. Age was significantly associated with scores of category switching fluency (*r* = −0.347, *P* = 0.001) and category switching accuracy (*r* = −0.361, *P* = 0.001), percent ratio of correct design (*r* = −0.352, *P* = 0.001), and sort recognition description (*r* = −0.321, *P* = 0.002). Age was positively correlated with color-word inhibition reaction times (*r* = 0.462, *P* < 0.001). Supplementary Figure S1 shows the scatter plots of age and these five D-KEFS scores.

**TABLE 2:** Descriptive analysis on D-KEFS scores, and the partial correlations of each D-KEFS scores and age, with gender, handedness, and body mass index (BMI) as covariates.

**Task**	**Scores**	**Range**	**Mean ± SE**	**Correlation with Age *R* (*P*)**
Verbal Fluency	Letter Fluency	18–67	42.67 ± 1.15	0.094 (0.382)
	Category Fluency	18–63	42.73 ± 0.95	−0.217 (0.042)
	Category Switching Fluency	7–20	13.17 ± 0.30	**−0.347 (0.001)**
	Category Switching Accuracy	3–19	11.41 ± 0.34	**−0.361 (0.001)**
Design Fluency	Empty Dots Design	2–18	10.65 ± 0.34	−0.139 (0.197)
	Filled Dots Design	4–20	10.27 ± 0.35	−0.113 (0.292)
	Alternating Design	14–50	32.63 ± 0.81	0.214 (0.045)
	Percent Ratio of Correct Design	34–100	82.49 ± 1.45	**−0.352 (0.001)**
Color Word Interference	Word Naming	12–33	20.66 ± 0.45	0.145 (0.177)
	Color Naming	16–45	27.12 ± 0.54	0.262 (0.014)
	Color-word Inhibition	26–95	50.88 ± 1.32	**0.462 (<0.001)**
Sorting	Free Sort Confirmed Sorts	2–53	9.60 ± 0.55	−0.167 (0.121)
	Recognition Sorts	16–63	36.73 ± 1.16	**−0.321 (0.002)**
	Description Sorts	8–59	34.66 ± 1.12	−0.205 (0.056)
Twenty Questions Tests	Total Questions Asked	16–46	26.62 ± 0.61	0.051 (0.634)
	Total Weighted Achievement	7–20	15.12 ± 0.28	−0.080 (0.457)
	Initial Abstraction	7–60	29.09 ± 1.31	−0.027 (0.801)

*Significant correlations were showed in bold fonts.*

### VMHC Results

**FIGURE 1:**
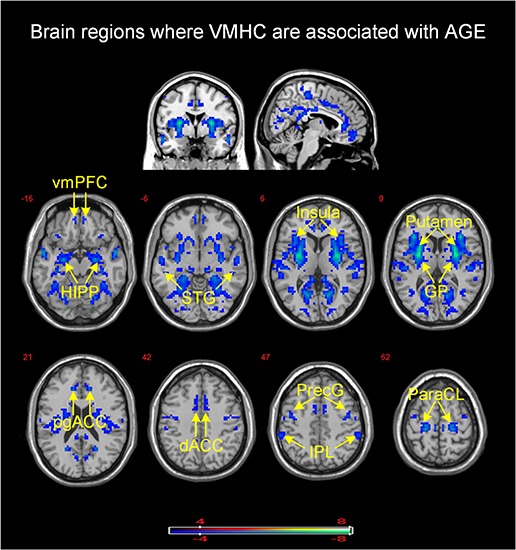
Brain mapping of VMHC demonstrated a significant association with age (*P*_FWE_ = 0.05, family-wise error correction). VMHC was negatively associated with age in brain regions with cool color. Color bar provides *T* values. vmPFC, ventromedial prefrontal cortex; HIPP, hippocampus; STG, superior temporal gyrus; GP, globus pallidus; pgACC, perigenual anterior cingulate cortex; dACC, dorsal anterior cingulate cortex; PreG, precentral gyrus; IPL, inferior parietal lobule; ParaCL, paracentral lobule.

There was no brain region in which VMHC correlated positively with age. Age correlated negatively with VMHC between bilateral putamen, insula (BA 13), hippocampus, superior temporal gyrus (BA 22), globus pallidus, paracentral lobule (BA 3), precentral gyrus (BA 4), perigenual and dorsal anterior cingulate gyrus (pgACC and dorsal ACC, BA 24), ventromedial prefrontal cortex (vmPFC, BA 10), and inferior parietal lobule (IPL, BA 39) (see Table 3 and Figure 1).

**TABLE 3:** The foci of brain areas showed intrinsic activity associating with age (*P*_FWE_ = 0.05, family-wise error correction) when controlling for gender, handedness, body mass index (BMI), FD, and total intracranial volume (TICV).

**Region**	**BA**	**Voxel**	***Z***	**MNI**		
				***X***	***Y***	***Z***
***Brain regions that VMHC showed negative correlation with age***						
Putamen	–	2733	7.08	± 27	−3	9
Insula	BA 13		6.02	± 36	21	6
Hippocampus	BA 28		5.72	± 24	−15	−21
Superior Temporal Gyrus	BA 22		5.42	± 54	−9	−15
Globus Pallidus	–		5.23	± 24	15	6
Paracentral Lobule	BA 3	129	5.53	± 21	−30	60
Precentral Gyrus	BA 4	131	4.94	± 45	−9	48
Anterior Cingulate	BA 24	149	4.96	± 12	33	21
Anterior Cingulate	BA24		4.35	± 6	6	42
Medial Frontal Gyrus	BA 10	36	4.48	± 6	54	−9
Medial Frontal Gyrus	BA 10		4.47	± 6	48	−18
Inferior Parietal Lobule	BA 40	47	4.22	± 60	−39	45

*BA, Brodmann area; –, no BA covered.*

### ROI-Based Analysis

CWIT inhibition reaction times correlated negatively with VMHC in the insula (*r* = −0.41, *P* = 0.00007), vmPFC (*r* = −0.46, *P* = 0.000006), hippocampus (*r* = −0.39, *P* = 0.0002), and superior temporal gyrus (*r* = −0.37, *P* = 0.0004). The percent ratio of correct design scores correlated positively with VMHC in the IPL (*r* = 0.36, *P* = 0.0006) (Supplementary Figure S2 shows scatter plots of the significant correlations above). The result showed that FCs_nolag was significantly larger than FCs_leftlag [*F*(1,20) = 204.208, *P* < 0.001] and FCs_rightlag [*F*(1,20) = 214.208, *P* < 0.001]. There was no significant difference between the FCs_leftlag and FCs_rightlag [*F*(1,20) = 1.987, *P* = 0.174].

### Mediation Analysis

**FIGURE 2:**
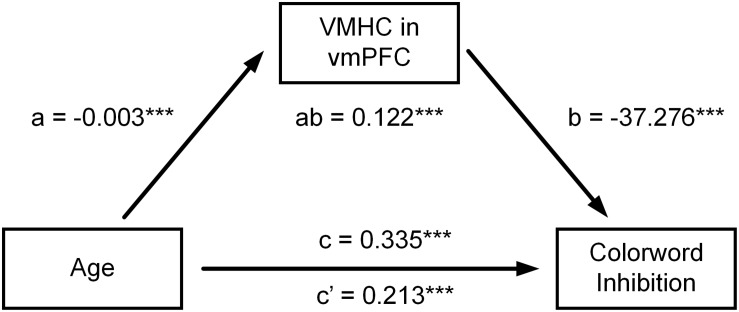
Mediation models on the relationship between age and CWIT inhibition reaction times. The relationship between age and CWIT inhibition reaction time was attenuated after controlling for VMHC of bilateral vmPFC. However, the direct effect of age on CWIT inhibition reaction time was still significant. Their relationship was therefore partly mediated by VMHC of bilateral vmPFC. ***P < 0.001.

Table 4 shows all estimated mediation models. When multiple comparisons were considered, mediation analyses showed that VMHC in vmPFC significantly mediated the relationship between age and CWIT inhibition reaction time (vmPFC: *ab* = 0.122, *P* = 0.0003, confidence interval (CI): [0.041, 0.262]) (see Figure 2).

**TABLE 4:** Mediation models on the relationship between age and D-KEFS scores.

**D-KEFS task *(Y)***	**Brain region *(M)***		**Path a (*X −* > *M*)**	**Path b (*M -* > *Y*)**	**Path c’(a − > Y)**	**Path c (*a -* > *Y*)**	**Mediation path *ab***
CWIT inhibition scores	Hippocampus (±24, −15, −21)	Beta	−0.005	−25.012	0.209	0.335	0.125
		*P*	0.0003	0.046	0.006	0.00003	0.026
		CI	[−0.007, −0.003]	[−48.716, −3.061]	[0.051, 0.394]	[0.181, 0.512]	[0.013, 0.277]
	Insula (±36, 21, 6)	Beta	−0.005	−27.078	0.206	0.334	0.128
		*P*	0.0003	0.032	0.007	0.00002	0.027
		CI	[−0.006, −0.003]	[−51.450, −2.091]	[0.057, 0.393]	[0.183, 0.518]	[0.015, 0.276]
	Precentral Gyrus (±45, −9, 48)	Beta	−0.004	−24.826	0.232	0.334	0.102
		*P*	0.001	0.048	0.003	0.00002	0.014
		CI	[−0.006, −0.003]	[−48.027, −4.394]	[0.082, 0.387]	[0.181, 0.510]	[0.016, 0.245]
	**vmPFC (±6, 48,**−**18)**	**Beta**	−**0.003**	−**37.276**	**0.213**	**0.335**	**0.122**
		*P*	**0.0009**	**0.0009**	**0.0005**	**0.00002**	**0.0003**
		**CI**	**[**−**0.005,**−**0.001]**	**[**−**57.702,**−**17.681]**	**[0.092, 0.356]**	**[0.180, 0.517]**	**[0.041, 0.262]**
The percent ratio of	Inferior Parietal Lobule (±60, −39, 45)	Beta	−0.004	29.821	−0.146	−0.251	−0.106
correct design scores		*P*	0.0001	0.002	0.105	0.0007	0.014
		CI	[−0.005, −0.002]	[10.700, 52.456]	[−0.332, 0.018]	[−0.422, −0.109]	[−0.221, −0.040]
	Anterior Cingulate Cortex (±12, 33, 21)	Beta	−0.003	−33.278	−0.350	−0.250	0.100
		*P*	3.04E-06	0.019	0.0006	0.001	0.015
		CI	[−0.004, −0.002]	[−60.171, −4.815]	[−0.545, −0.182]	[−0.424, −0.105]	[0.022, 0.206]
	Globus Pallidus (±24, 15, 6)	Beta	−0.004	−41.254	−0.407	−0.251	0.156
		*P*	0.0001	0.002	0.0004	0.001	0.016
		CI	[−0.005, −0.003]	[−69.503, −5.897]	[−0.610, −0.183]	[−0.430, −0.107]	[0.028, 0.290]
	Putamen (±27, −3, 9)	Beta	−0.005	−29.378	−0.400	−0.251	0.149
		*P*	0.0009	0.023	0.001	0.002	0.025
		CI	[−0.006, −0.004]	[−53.794, −3.423]	[−0.646, −0.162]	[−0.423, −0.106]	[0.021, 0.295]
Sort recognition	Hippocampus (±24, −15, −21)	Beta	−0.005	−20.805	−0.266	−0.168	0.098
description scores		*P*	0.0002	0.020	0.0004	0.013	0.015
		CI	[−0.006, −0.003]	[−40.696, −2.551]	[−0.410, −0.123]	[−0.295, −0.037]	[0.019, 0.210]
	Putamen (±27, −3, 9)	Beta	−0.005	−27.758	−0.307	−0.168	0.139
		*P*	0.001	0.012	0.0003	0.0166	0.005
		CI	[−0.006, −0.004]	[−49.604, −7.309]	[−0.481, −0.143]	[−0.302, −0.040]	[0.039, 0.262]

*In the model, age was the independent variable, each D-KEFS score was set to be the dependent variable. VMHC of brain regions significantly associated with age and D-KEFS score was set to be mediator, respectively. Path “a” was the effect of independent variable on mediator, Path “b” was the effect of mediator on dependent variable with independent variable as covariates. Path “c”’ was the direct effect of independent variable on dependent variable with mediator being taken into consideration. Path “c” was the direct effect of independent variable on dependent variable without mediator being taken into consideration. Mediation path “ab” was the effect of mediator on the relationship of the independent variable on the dependent variable. Gender, handedness, body mass index (BMI), FD, and total intracranial volume (TICV) were included as covariates. CI, confidence interval. The model with significant mediation effect was showed in bold fonts.*

At an uncorrected level, VMHC in insula, hippocampus, and precentral gyrus also mediated the relationship between age and CWIT inhibition reaction time (insula: *ab* = 0.128. *P* = 0.027, CI: [0.015, 0.276]); hippocampus: *ab* = 0.125. *P* = 0.026, CI: [0.013, 0.277]; precentral gyrus: *ab* = 0.102. *P* = 0.014, CI: [0.016, 0.245]). Moreover, VMHC in IPL, pgACC, globus pallidus, and putamen mediated the relationship between age and the percent ratio of correct design scores, respectively (IPL: *ab* = −0.106, *P* = 0.014, CI: [−0.221, −0.040]; pgACC: *ab* = 0.100, *P* = 0.015, CI: [0.022, 0.206]; globus pallidus: *ab* = 0.156, *P* = 0.016, CI: [0.028, 0.290]; putamen: *ab* = 0.149, *P* = 0.025, CI: [0.021, 0.295]).

## Discussion

Little is known regarding the influence of age on brain homotopic functional coupling and how this reorganization might be associated with age-related executive function alterations. In line with FC-based rs-fMRI studies on normal aging, here we observed age-associated reduction of interhemispheric FC of medial temporal lobe subsystem (vmPFC and pgACC and hippocampus) within DMN, dACC and insula within SN, and IPL within FPCN. Further, we showed that response inhibition performance was associated with reduced interhemispheric functional coupling of medial temporal lobe subsystem of DMN and insula in SN, and correct design scores were associated with functional coupling of bilateral IPL of FPCN. Our findings extend previous studies on age-related intrinsic functional network reorganization (Damoiseaux et al., 2008; Keiichi et al., 2012; Chan et al., 2014; Grady et al., 2016) and indicate that the alterations of interhemispheric functional coupling might also at least partly underlie age-related executive function change.

### Age-Related Executive Function Alterations

In the current study, age was negatively associated with performance in color-word inhibition (reaction time), category switching task (fluency and accuracy), design fluency (percent ratio of correct design), and sort recognition tasks. Performance on these tasks assesses higher executive function, such as inhibitory control, cognitive flexibility, and conceptual reasoning. The previous cross-sectional (Ivnik et al., 1996; Klein et al., 1997; Harrington et al., 2017) and longitudinal studies (Adólfsdóttir et al., 2016) of the Stroop task have indicated a negative relationship between response inhibition and age after controlling for basic word naming and color naming conditions. This negative relationship between category switching performance and age seems to corroborate the previous study indicating significant age-related effects in category switching task (Wecker et al., 2005; Lanting et al., 2009). Moreover, older adults were found to complete significantly fewer designs than middle-aged adults in design fluency task (Ready, 2010; Sanders and Schmitter-Edgecombe, 2012). Age has also been negatively associated with scores of sort recognition (Mattioli et al., 2014). These results reiterate the viewpoint that higher executive function is vulnerable to aging.

### Age-Related Interhemispheric FC Alterations

In the current study, age correlated negatively with VMHC of vmPFC, pgACC, and hippocampus areas that belong to the medial temporal lobe subsystem of DMN (Andrews-Hanna et al., 2010). The medial temporal lobe subsystem has been associated with episodic judgments about the personal future, among other high-level executive functions (Andrews-Hanna et al., 2010). Damoiseaux et al. (2008) have employed ICA and showed that network covariation of the anterior part of DMN inversely correlated with age. Further, FC density of the vmPFC is decreased with age (Tomasi and Volkow, 2012). Previous FC studies have indicated that the DMN may be one of the brain networks most vulnerable to aging. Our results thus extend prior work indicating that age affects the interhemispheric functional coupling of the medial temporal lobe subsystem of DMN.

We observed that VHMC of bilateral dACC and insula attenuated with aging, both of which were key components of SN. The insula is involved in detecting and selecting salient stimuli by combining endogenous and exogenous information, and mediating interactions with other neurocognitive systems (Seeley et al., 2007; Tim et al., 2007; Sridharan et al., 2008). A body of studies have shown reduction of intra-network FC of dACC and insula with aging (Keiichi et al., 2012; He et al., 2014; Zhang et al., 2014). The inter-network FC profiles between SN and visual network, as well as the SN and the anterior part of the DMN, are powerful predictors of age (Keiichi et al., 2012). These results are in line with the notion that SN is one of the brain networks most vulnerable to aging (La Corte et al., 2016). However, relatively few studies have examined the interhemispheric FC strength of SN. There is one study showing that FC strength of the bilateral insula negatively correlates with age (Keiichi et al., 2012). Our findings confirm these results, and further indicate that interhemispheric functional coupling of dACC decrease with age increase, suggesting that these effects extend to other nodes of the SN.

In the current study, VMHC of IPL (belonging to FPCN) correlated negatively with age. IPL is involved in adaptive cognitive control decision-making processes (Vincent et al., 2008). With regard to neurocognitive aging, IPL has shown an asymmetric response pattern in cognitive tasks for older and younger adults: younger adults utilize the left IPL more than older adults when ignoring irrelevant stimuli on 1-back memory task (Campbell et al., 2012). However, older adults show stronger activation of the right IPL during target detection than young adults (Geerligs et al., 2014b). These two studies may emphasize the adapting response of IPL to external cognitive control task for older adults. Grady et al. (2016) have employed a graph theory method to show that, in older adults, the bilateral IPL are functionally stronger connected with brain cortices in the dorsal attention network than with classic brain regions in FPCN, indicating the functional reorganization of parietal regions for older adults. The current study added to this literature in that IPL showed reorganization of interhemispheric functional coupling in older adults. However, Madhyastha and Grabowski, 2014 find that the FC between bilateral IPL is not correlated with age in older subjects (aged 56–89), possibly because FC between bilateral IPL is vulnerable to age at an early stage (Siman-Tov et al., 2017).

### The Relationship of Age-Related VMHC Alterations and Executive Function

CWIT inhibition scores (reaction time) correlated negatively with VMHC in vmPFC. The vmPFC is implicated in governing goal-directed learning (Valentin et al., 2007; Sanne et al., 2009) and decision-making (Sanne et al., 2009; Reber et al., 2017) for outcome valuation. Previous fMRI studies have also suggested that vmPFC is more heavily recruited during the processing of incongruent trials in a spatial Stroop task, and its activation has shown correlations with the efficiency of top-down cognitive control (Araneda et al., 2018). fMRI studies have documented an association between diminished activity in vmPFC and poor performance on the Stroop task in pathological gamblers (Potenza et al., 2003) and individuals with binge eating disorder (Balodis et al., 2013), suggesting a pivotal role of vmPFC in cognitive control. In line with this, the negative association between VMHC of vmPFC and CWIT inhibition reaction times confirms the key role of vmPFC in cognitive control, suggesting that the functional coordination of vmPFC is also a sensitive neural marker to age-related change in response to inhibition performance. One possible explanation is that inhibition requires a decision that is congruent on a complex goal rather than an immediate response to stimuli; this process may depend on the encoding of goal values by vmPFC (Reber et al., 2017). In addition, we showed that VMHC of bilateral vmPFC mediated the influence of age increase on CWIT inhibition reaction time, indicating that age-related functional alterations of vmPFC might be part of the neural mechanism underlying age-related decline of response inhibition (Andrews-Hanna et al., 2007).

CWIT inhibition reaction time also correlated with VMHC of the bilateral insula. The right insula has been emphasized by its prominent role in saliency processing and initiating attentional control during executive control behaviors (Eckert et al., 2009, 2010). The bilateral insula have been indicated as common regions that are recruited in Go/NoGo, Flanker and Stimulus-response compatibility tasks (Morelli et al., 2015). Their task-related activation levels are shown to correlate with behavioral performance (Eckert et al., 2010; Morelli et al., 2015), such as Stroop performance (Leung et al., 2000; Potenza et al., 2003; Zysset and Schroeter, 2007; Balodis et al., 2013). One rs-fMRI study finds a positive association between FC of the bilateral insula with categorical verbal fluency test scores (Keiichi et al., 2012). In line with these studies, our findings show that insular VMHC numerically mediated the relationship between age and CWIT inhibition performance, suggesting that functional coupling of bilateral insula is, at least partly, involved in neural mechanisms underlying age-related decline in response inhibition.

In the current study, percent ratio of correct design scores correlated positively with VMHC of IPL. A body of literature has implicated that IPL is commonly involved in executive function tasks, such as working memory, response inhibition, interference control, and sustained attention (Langner and Eickhoff, 2013; Cieslik et al., 2015; Krall et al., 2015). Moreover, an accumulating literature has shown that performance in design fluency task is positively associated with bilateral IPL gray matter volume (Kramer et al., 2007; Possin et al., 2012). Furthermore, interhemispheric IPL connectivity has shown to be reduced in patients after pediatric arterial ischemic stroke, and D-KEFS category fluency correlated positively with the interhemispheric IPL connection in both these patients and healthy controls (Kornfeld et al., 2018). In line with the study above, we also found that age-related reductions in connectivity of bilateral IPL contributed to attenuation of performance in design fluency task. Successful design fluency task complement has been shown to require processing of abundant bottom visual and motor information under top-down cognitive control (Yana et al., 2010). IPL comprises multimodal neurons for integration of auditory, sensory, visual, and motor information (Daniel et al., 2015) and receipt top-down regulation of dACC for cognitive control (Harding et al., 2015), which is a candidate brain region to be involved in design fluency task. In line with this point, VMHC of IPL mediated the relationship between age and the percent ratio of correct design scores (at trend level), suggesting that IPL function plays a pivotal role in age-related performance alteration of design fluency task. Our findings extend previous ones that found age-related functional reorganization of FPCN contributing to cognitive ability decline, suggesting that the alterations of interhemispheric functional coupling of posterior parietal cortex might also contribute to age-related performance decline in design cognition.

### Limitations

In the current study, there are some limitations that should be noted. First, previous studies have indicated that aging influences the brain in multiple ways, such as alteration of cortical thickness, gray matter volume, and functional organization. The current study shed light on alterations of interhemispheric communication with aging, but in future work, it will be important to study other kinds of brain FC to investigate more comprehensive patterns of age-related brain functional reorganization. For example, one could employ connectome-based individualized prediction modeling (Jiang et al., 2018). Second, we did not perform test–retest examination on independent data due to unavailability of a similar resting fMRI data with similar age range and executive function test. Future studies are thus needed to replicate our findings.

## Conclusion

The current study highlights age-related interhemispheric FC alterations and explores the links between these alterations and executive function. Aging was associated with interhemispheric FC alterations of brain areas belonging to medial temporal lobe subsystem of DMN, insula, and dorsal ACC of SN, and IPL of FPCN. Further, interhemispheric FC alterations contributed to age-related executive function change. Our findings provide new evidence for theories of age-related cognitive decline.

## Data Availability Statement

The datasets generated for this study are available on request to the corresponding author.

## Ethics Statement

The studies involving human participants were reviewed and approved by the local Institutional Review Board (IRB) at the NKI. The patients/participants provided their written informed consent to participate in this study.

## Author Contributions

JZ, HS, PZ, JG, and YS analyzed the data and performed the statistical analysis. JZ, PM, CW, and G-JW wrote the first draft of the manuscript. G-JW and DH contributed to the conception and design of the study.

## Conflict of Interest

The authors declare that the research was conducted in the absence of any commercial or financial relationships that could be construed as a potential conflict of interest.

## Footnotes

1 http://www.fil.ion.ucl.ac.uk/spm
