# Supplementary material for: Age-Related Decreases in Interhemispheric Resting-State Functional Connectivity and Their Relationship With Executive Function
Source: Front Aging Neurosci. 2020 Feb 26;12:20. doi: 10.3389/fnagi.2020.00020 (PMC7054233; doi:10.3389/fnagi.2020.00020)
Supplement: Supplementary file 1 [file Data_Sheet_1.pdf]

Supplementary Material

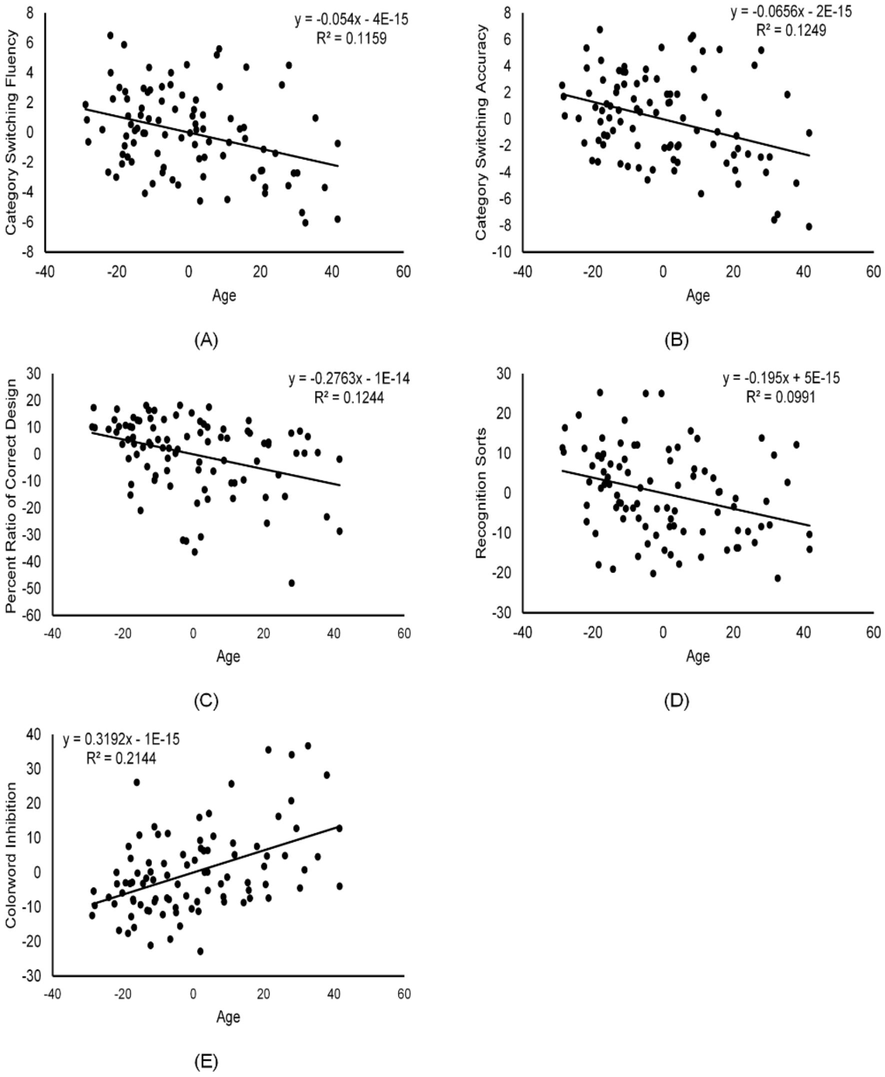

Supplemental Figure 1 Scatter plots of each D-KEFS scores and age. Gender, handedness and BMI was regressed out. (A) Scatter plots of category switching fluency scores and age. (B) Scatter plots of category switching accuracy scores and age. (C) Scatter plots of percent ratio of correct design scores and age. (D) Scatter plots of sort recognition scores and age. (E) Scatter plots of CWIT-inhibition reaction time scores and age.

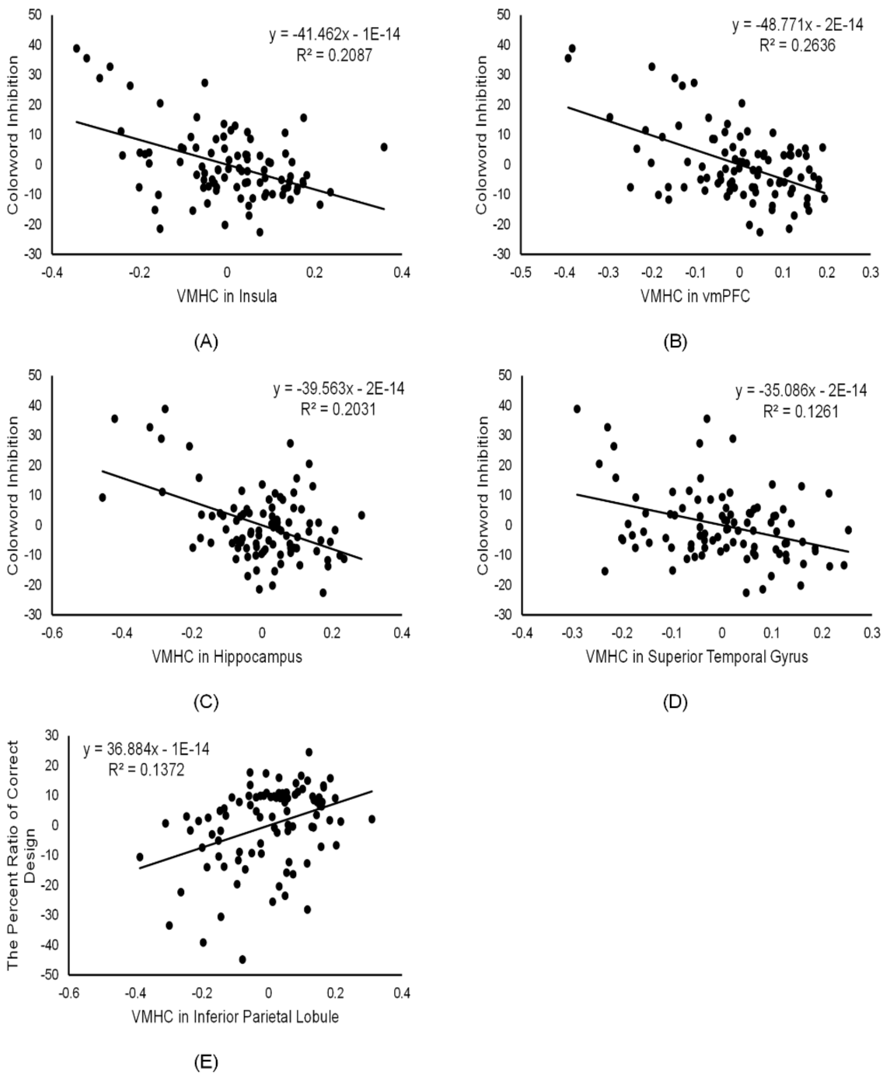

Supplemental Figure 2 Scatter plots of D-KEFS scores and brain regions which VMHC was significantly associated with age. Gender and handedness, Body mass index (BMI), FD and total intracranial volume (TICV) were regressed out. (A) Scatter plots of CWIT-inhibition reaction time scores and VMHC in insula. (B) Scatter plots of CWIT-inhibition reaction time scores and VMHC in vmPFC. (C) Scatter plots of CWIT-inhibition reaction time scores and VMHC in hippocampus. (D) Scatter plots of CWIT-inhibition reaction time scores and VMHC in superior temporal gyrus. (E) Scatter plots of percent ratio of correct design scores and VMHC in inferior parietal lobule.
